# Relationship between male moths of *Cryptoblabes gnidiella* (Millière) (Lepidoptera: Pyralidae) caught in sex pheromone traps and cumulative degree-days in vineyards in southern Uruguay

**DOI:** 10.1186/2193-1801-2-258

**Published:** 2013-06-10

**Authors:** María Valeria Vidart, María Valentina Mujica, María Victoria Calvo, Felicia Duarte, Carlos María Bentancourt, Jorge Franco, Iris Beatriz Scatoni

**Affiliations:** Department of Plant Protection, Faculty of Agronomy, University of the Republic, Ave. E. Garzón 780, Montevideo, 12900 Uruguay; Department of Biometry, Statistics and Computation, Faculty of Agronomy, University of the Republic, Ave. E. Garzón 780, Montevideo, 12900 Uruguay

**Keywords:** Honeydew moth, Flight activity, Hibernation, Degree-day models

## Abstract

*Cryptoblabes gnidiella* (Millière) (Lepidoptera: Pyralidae) has been known in Uruguay for 30 years and only in vineyards, despite being polyphagous. In recent years, this pest has caused sporadic but serious damage on some grapevine cultivars. Understanding the insect’s phenology and developing a monitoring program are essential aspects of integrated pest management. We monitored males using sexual pheromone traps on four cultivars of vine, Pinot noir, Tannat, Gewürztraminer, and Cabernet Sauvignon, in two vine-growing establishments in the Department of Canelones and compiled data on the accumulated effective temperatures for the southern area of Uruguay. We determined that this species undergoes three generations per year and overwinters without diapause as larvae on dried grapes remaining after harvest. Using the proportion of cumulative male moths caught from December to May from 2003–2007 on the four cultivars and the sum of effective temperatures above two previously-published lower-threshold temperatures for development, 12.26°C and 13°C, statistically significant logistic models were estimated. Predictions based on the resulting models suggested that they would be acceptable tools to improve the efficiency of integrated management of this pest in Uruguay.

## Background

In Uruguay, vineyards have undergone sustained plant replacement. In the past 20 years, most of the country's 8,000 hectares of vineyards have been replaced by newer, healthier, and higher-quality grapevine cultivars. Eighty-nine percent of the wine growing area is concentrated in southern Uruguay, especially in Canelones Department (MGAP–DIEA 2011). Unlike in other wine-producing areas of the world, pests have been a minor problem in Uruguay's vineyards, eliminating the need for widespread applications of insecticides (Bentancourt and Scatoni 1999).

*Cryptoblabes gnidiella* (Millière) (Lepidoptera: Pyralidae) has become a sporadic pest in Uruguay capable of causing significant damage to some grapevine cultivars in certain years and areas (Bentancourt and Scatoni 2006). This polyphagous moth, is native to the Mediterranean regions of Europe and reported from Africa, Asia, New Zealand, North and South America (Bagnoli and Lucchi 2001, Ioriatti *et al.*^2012^). It has been known in Uruguay for 30 years, but only reported from vineyards (Scatoni and Bentancourt 1983). Since its appearance, it has displaced in importance two other grape pests: *Argyrotaenia sphaleropa* (Meyrick) and *Bonagota salubricola* (Meyrick) (Lepidoptera: Tortricidae). The larvae feed on grape cluster, especially at the end of season when the fruits are already mature. Feeding damage produces conditions conducive to the development of rots. The economic losses become more significant when harvest is delayed, due to an increase in population and a potential additional generation. Also, rainfall and high humidity create conditions suitable for rots causing further deterioration of the clusters (Bentancourt and Scatoni 2006).

Knowing pest phenology is an essential aspect of developing a management program. The identification of the sex pheromone of *C. gnidiella* provided a monitoring tool for adults now widely used (Bjostad *et al.*^1981^, Anshelevich *et al*. 1993). Monitoring of adults as well as degree-days (DD) allows the prediction of pest phenological events for management purposes. Numerous reports have correlated species catches with DD for several species of Lepidoptera; as an example, these relationship have been studied for *Lobesia botrana* (Lepidoptera: Tortricidae), the main vineyard pest in Europe (Del Tio *et al.*^2001^, Milonas *et al.*^2001^). The thermal constant and lower thresholds of development for *C. gnidiella* were determined by Avidov and Gothilf (1960) for Israel and by Ringenberg *et al.* (2005) for Brazil. There is, however, no information available about the relationship between DD and catch levels.

Understanding a pest's phenology and monitoring its populations are essential aspects of integrated pest management. The objective of this research was to better understand the phenology of this insect in Uruguay and the damage it inflicts on cultivars with different maturity dates to develop a forecasting system that uses pheromone traps and the accumulation of effective temperatures. For these purposes, population’s growth models were run for each cultivar and for all cultivars.

## Materials and methods

### Study sites

The study was carried out in two vineyards, 10 km apart, in Canelones Department, which represents our country's wine production. One was located in Juanicó (34°58'S, 56°25'W) and the other in Progreso (34°68'S, 56°21'W). The first being a 200 hectares vineyard and the second 50 hectares. Both vineyards have several cultivars with varying harvest dates, ranging from mid-February to mid-April. Vineyard design is presented in Table 1. The vineyard was managed without irrigation and in all cases with resident inter-row vegetation and herbicides along the row. In neither vineyard were insecticides applied during the study period, from 2003–2007.

**Table 1:** **Vineyard design at Progreso and Juanicó, Uruguay**

Site	Cultivar	Rootstock	Year planted	Planting distance (m)	Conduction system
Progreso	Cabernet Sauvignon	SO4	1986	3.00 × 1.00	lyre
	Pinot noire	SO4	1994	3.00 × 1.25	lyre
Juanicó	Tannat	SO4	1994	3.00 × 1.25	lyre
	Gewürztraminer	SO4	1986	3.00 × 1.00	lyre

### Monitoring adults

Adult populations were monitored with delta traps baited with 1 mg of synthetic sex pheromone (Z11-16: Ald and Z13-18: Ald, 1:1, Yogev® Ltd, Rishon Le’zion, Israel). Traps were placed 1.5 m above the ground and checked weekly. In Juanicó, three traps were placed 200 m apart on Pinot noir, Tannat, and Gewürztraminer cultivars and were monitored from December 2003 to June 2007. In Progreso, one trap was placed on Cabernet Sauvignon from October 2004 to June 2007. Each cultivar occupied an area of about one hectare and the trap was placed in the middle of the plot. Pheromone lures were replaced weekly and sticky bottom whenever necessary.

### Monitoring larvae

Larvae were monitored from December until leaf fall. For each of the cultivars where adult traps were placed, 90 clusters (two per vine) were collected at random every 2 weeks. In the laboratory, we recorded the presence of damage and the number of larvae and pupae per cluster. Insects collected were stored in boxes with the clusters and kept until either adults or parasitoids emerged. To understand the behavior of the overwintering larvae, after harvest we collected 60 infested clusters per year from each cultivar and stored them in 25 × 30 cm netting cloth bags in the laboratory for 24 h. The bags were returned to the vineyard the following day and hung from the wires of the lyre. Bags were checked every fortnight to verify larval development and adult emergence per cultivar. In addition, during the plants’ dormancy period we directly observed beneath the rhytidome and in other places where the larvae might be (leaf litter, dead leaves).

### Phenological models

Daily maximum and minimum temperatures were taken from the Experimental Station of National Agricultural Research Institute Las Brujas from 2003–2007. This station is located 10 and 12 km, respectively, from the Progreso and Juanicó vineyards. Degree-days were estimated using the Baskerville and Emin (1969) method based on maximum and minimum air temperature.

To estimate the mean generation time under field conditions, we used the cumulative sum of effective temperatures (DD) between the start of one generation's flight and the next. The beginning of the overwintering flight was taken as the first date on which male moths were caught on consecutive days; this occurred in early December in all 4 years of the study. For this reason we used December 1^st^ as the biofix. A similar method was used to set the biofix of *Cydia pomonella* ( Riedl *et al.*^1976^) and other Tortricidae (Knight and Croft 1991). To set the start of subsequent generations, we used the dates on which number of male moths caught were high after periods of consistently declining or zero catches. According to Avidov and Gothilf (1960), *C. gnidiella* requires a minimum temperature of 13°C for development and 500 DD to complete a generation. However, Ringenberg *et al.* (2005) suggest a lower threshold of development of 12.26°C and 570 DD to complete a generation.

Mathematical models were adjusted using the accumulation of DD and the proportion of cumulative catches at the two sites for the 4 years and four grapevine cultivars. These models can be used to predict how the population will develop as a function of DD accumulated over time. We used one logistic model: *logit* (*p*) = *a* + *bx;* where *logit* (*p*) = *log* (*p* / (1–*p*)), *p* is the cumulative proportion of adult males associated with *x, a* and *b* are parameters of the model, and *x* is the cumulative DD. Estimation was done in the framework of generalized linear models (McCullagh and Nelder 1999) assuming a binomial distribution and a logit link function. To test the hypotheses of equality of the model parameters, we compared confidence limits, (when two intervals overlapped, the parameters were considered to be equal; otherwise they were defined as different). Finally, to compare the averages of weekly catches, we applied likelihood ratio and Tukey–Kramer tests. These comparisons of mean values were made in framework of the generalized linear model (McCullagh and Nelder 1999). Analyses were done using the GLIMMIX procedure in SAS v. 9.2 (SAS Institute Inc 2009).

## Results

### Monitoring adults

**Figure 1:**
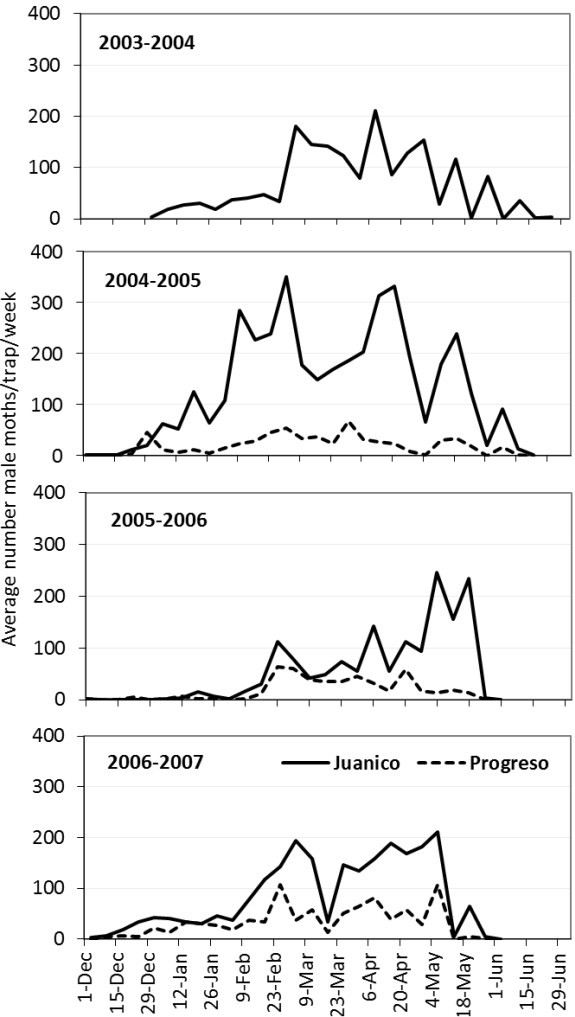
**Average number of male of*Cryptoblabes gnidiella*caught in pheromone traps in vineyards at Juanicó and Progreso, Uruguay.**

Changes in the *C. gnidiella* populations in vineyards based on adult monitoring with pheromone traps from 2003–2007 are presented in Figure 1. Adults that fly in early December belong to the overwintering generation, which lasts until late January–early February. The first generation develops during February and March, the second and final generation develops primarily after harvest and the females lay eggs in remaining clusters.

Thermal requirements starting from biofix (December 1^st^) also identified three generations. We use 13° and 12.26°C as lower-threshold temperatures and 500 and 570 DD, respectively, as thermal constants. Table 2 shows the starting dates and accumulated DD of the first and second generations in different years, using the temperature thresholds above mentioned.

**Table 2:** **Degree-days accumulated by generation of*Cryptoblabes gnidiella*in Uruguay from 2003–2007**

Year	Start date^1^	DD accumulated	DD accumulated	Start date^1^	DD accumulated	DD accumulated
	1^st^ generation	LTT 13°C^2^	LTT 12.26°C^3^	2^nd^ generation	LTT 13°C^2^	LTT 12.26°C^3^
2004	29-Jan	482	527	01-Apr	999	1090
2005	27-Jan	527	570	31-Mar	1009	1098
2006	02-Feb	489	536	13-Apr	989	1089
2007	22-Jan	500	540	19-Mar	1011	1092
Mean		500 ± 20	543 ± 19		1002 ± 10	1092 ± 04

^1^Start date of each generation was determined from catches of males in pheromone traps, ^2^lower-temperature thresholds determined by Avidov and Gothilf (1960), ^3^lower-temperature thresholds determined by Ringenberg *et al*. (2005).

In Juanicó, number of male moths caught in traps was always higher than in Progreso, and there was also a year effect, as indicated by the higher 2004–2005 catches for all cultivars in Juanicó (Table 3). However, no differences were found among cultivars within a growing season at this site.

**Table 3:** **Average number of male moths of*Cryptoblabes gnidiella*caught in pheromone traps at two sites in Uruguay**

Site	Grapevine cultivar	Average number of male moths/trap/week^1^							
		2003–2004		2004–2005		2005–2006		2006–2007	
Juanico	Pinot noir	55.9	cde	103.7	ab	33.0	efg	42.5	def
Juanico	Tannat	58.0	cde	92.7	abc	34.3	efg	70.7	bcd
Juanico	Gewürztraminer	60.5	bcde	118.0	a	57.1	cde	72.2	bcd
Progreso	Cabernet Sauvignon	---		15.8	g	13.0	g	23.9	fg
Mean^2^				61.6	***a***	28.7	***c***	45.2	***b***

^1^ Means in the table followed by the same letter were not significantly (*p* ≤ 0.05) different according to the Tukey–Kramer test.

^2^ Means in the summary line followed by the same letter in italics were not significantly (*p* ≤ 0.05) different according to the Tukey–Kramer test.

### Monitoring larvae

The first larvae on clusters of grapes were found in mid-January, and damage began to be significant at the end of February. No damage was observed on Pinot noir because it is harvested in late January or early February. However, the larvae were abundant from late February in clusters remaining on the plants after harvest. On Tannat, the damage was slight, with 4–10% of clusters infested, depending on the year, but 50–83% of Gewürztraminer clusters were affected at harvest time (Table 4).

**Table 4:** **Percentage of clusters damaged by*Cryptoblabes gnidiella*on different grapevine cultivars in Uruguay at harvest time**

Site	Grapevine cultivar	Percent cluster damage				Harvest dates
		2004	2005	2006	2007	
Juanicó	Pinot noir	0	0	0	0	29-Jan to 5-Feb
Juanicó	Tannat	6	10	5	4	5 to 15-March
Juanicó	Gewürztraminer	81	83	50	63	5 to 19-March
Progreso	Cabernet Sauvignon	--	0	8	6	12 to 20-March

In Progreso, damage on Cabernet Sauvignon and number of male moths caught were very low throughout the season, although this cultivar is harvested on mid-March (Table 4).

The average number of larvae per infested cluster was two on Tannat, five on Cabernet Sauvignon, and four on Gewürztraminer. Larvae were more abundant close to harvest, however at that time, a single larva was enough to degrade the cluster quality, due to colonization of fungi that cause rot. The maximum number of larvae found on a cluster was 85, on 5 March 2005 on Gewürztraminer. In no case did parasitoids emerge from larvae or pupae collected in the field and reared individually in the laboratory.

Larvae and pupae overwinter under the rhytidome or in clusters and dry leaves that persist on the plant and develop slowly due to cold temperatures. Some adults emerged sporadically inside the cloth bags, but most did so when the traps registered the first catch.

### Phenological model

**Figure 2:**
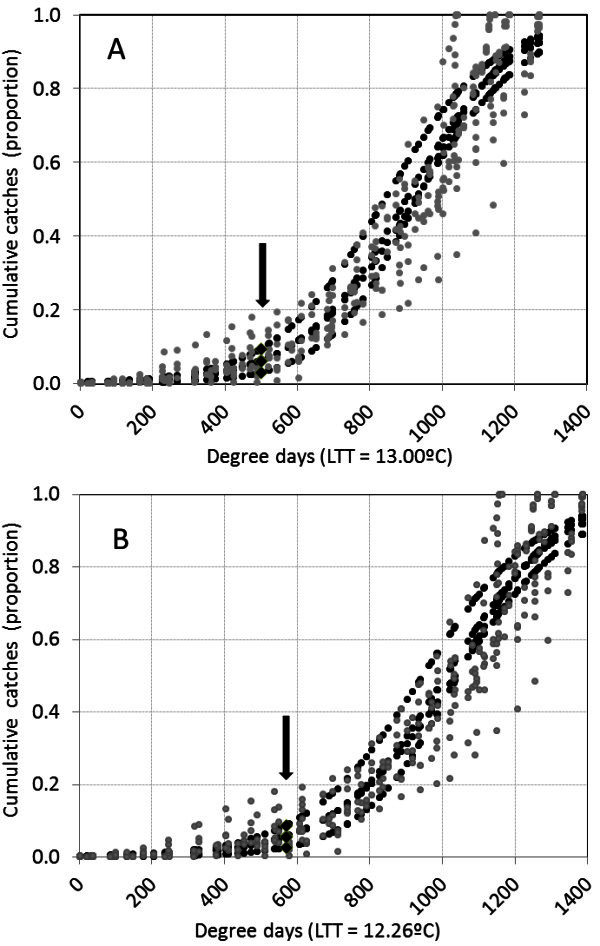
**Relationship between degree-days and the cumulative proportion of adult males of*Cryptoblabes gnidiella*caught in pheromone traps on each of four grapevine cultivars between 1**^**st**^**December and 31**^**st**^**May in 4 years and at two sites in Uruguay. A**) Employs the lower-temperature threshold determined by Avidov and Gothilf (1960); **B**) uses the lower-temperature threshold estimated by Ringenberg *et al.* (2005). Observed values are grey, estimated values are black, and estimated proportions at the end of the first generation are identified with an arrow.

The logistic models, estimated as  related the proportion of cumulative male moths caught in pheromone traps (*p*) for each grapevine cultivar to the accumulated DD in each year using the lower-threshold temperature proposed by Avidov and Gothilf (1960) and by Ringenberg *et al.* (2005). Table 5 presents the estimates of the *a* and *b* parameters for the models and the confidence intervals for the population growth rate (*b*). The corresponding graphs are in Figure 2.

**Table 5:** **Logistic models estimated for each grapevine cultivar and for all cultivars in a joint model**

Grapevine cultivar	LTT 13°C^1^					LTT 12.26°C^2^				
	***a***	***b***	95% Confidence limits			***a***	***b***	95% Confidence limits		
			LL	UL				LL	UL	
Pinot noir	-5.8436	0.0063	0.0038	0.0088	a†	-6.1673	0.0059	0.0036	0.0083	a
Tannat	-7.4594	0.0080	0.0047	0.0112	a	-7.9213	0.0076	0.0045	0.0107	a
Gewürztraminer	-6.1224	0.0068	0.0040	0.0095	a	-6.4848	0.0064	0.0038	0.0090	a
C. Sauvignon	-5.5514	0.0066	0.0037	0.0095	a	-5.9016	0.0062	0.0035	0.0090	a
***Joint model***	-6.1559	0.0068	0.0054	0.0082		-6.5190	0.0064	0.0051	0.0078	

The models relate the proportion of cumulative catches in pheromone traps to the accumulated degree-days in each year using the lower-temperature thresholds (LTT) ^1^determined by Avidov and Gothilf (1960) ^2^determined by Ringenberg *et al.* (2005), LL, confidence lower limit, UL, confidence upper limit ***a*** and ***b*** parameters estimated for the logistic models, .

†: Same letter indicates non-significant differences between ***b*** values (p ≤ 0.05) for the different grapevine cultivars.

The proportion of cumulative catches and DD were significantly correlated, regardless of the temperature threshold used. Adjusted models for each grapevine cultivar did not differ statistically from each other, both when comparing the growth rate of the logistic model (parameter *b*, Table 5) and when comparing the estimated proportion of accumulated catches at the average DD values (Table 6). For this reason we decided to use a joint model for all four grapevine cultivars and all 4 years of the study (Table 5).

**Table 6:** **Estimated proportions of cumulative catches for the average DD values of the logistic curve for four grapevine cultivars in Uruguay and at the end of the first generation in the joint model**

***Models for each cultivar***	LTT 13°C^1^				LTT 12.26°C^2^			
	Average 742 DD				Average 833 DD			
	Estimated proportion	95% Confidence limits			Estimated proportion	95% Confidence limits		
		LL	UL			LL	UL^2^	
Pinot noir	0.2362	0.1230	0.3786	a	0.2194	0.1171	0.3733	a
Tannat	0.1676	0.0747	0.3343	a	0.1587	0.0684	0.3263	a
Gewürztraminer	0.2395	0.1303	0.3985	a	0.2308	0.1228	0.3914	a
C. Sauvignon	0.3247	0.1843	0.5057	a	0.3166	0.1765	0.5003	a
All cultivars	0.2475	0.1840	0.3242		0.2383	0.1751	0.3154	
***Joint model***	**LTT 13°C**				**LTT 12.26°C**			
	**End of the first generation 500 DD**				**End of the first generation 570 DD**			
	**Estimated proportion**	**95% Confidence limits**			**Estimated proportion**	**95% Confidence limits**		
		**LL**	**UL**			**LL**	**UL**	
All cultivars	0.0597	0.0321	0.1082		0.0545	0.0286	0.1015	

^1^LTT lower-temperature thresholds determined by Avidov and Gothilf (1960), ^2^LTT lower-temperature thresholds determined by Ringenberg *et al*. (2005), LL, confidence lower limit, UL, confidence upper limit.

†: Same letter indicates non-significant differences among means (*p* ≤ 0.05).

**Figure 3:**
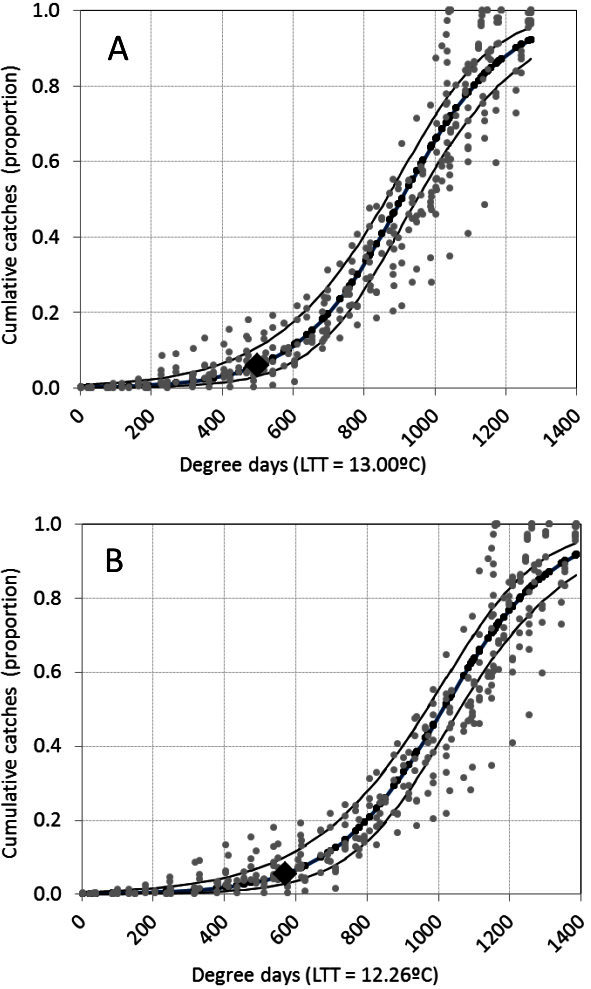
**Relationship between degree-days and the cumulative proportion of adult males of*Cryptoblabes gnidiella*caught in pheromone traps on four grapevine cultivars between 1**^**st**^**December and 31**^**st**^**May in 4 years and at two sites in Uruguay. A**) Employs the lower-temperature threshold determined by Avidov and Gothilf (1960); **B**) uses the lower-temperature threshold estimated by Ringenberg *et al.* (2005). Observed values are grey, estimated values are black, lines are 95% confidence limits, and ♦ is the estimated proportion at the end of the first generation.

Based on the adjusted models, we estimated the proportion of catches that would be achieved at the end of the first generation, which does not cause economic damage, and we were able to predict their magnitude at harvest for each cultivar (Figures 3). According to the models, the first generation ended at 500 or 570 DD, when the proportion of captured adults was 0.06 and 0.05 respectively (Table 6). In our study, the cumulative catches at that time were 326 males in Juanicó and 80 in Progreso in 2005. Depending on the year, the harvests of Tannat, Gewürztraminer, and Cabernet Sauvignon took place between 5 to 20-March, the cumulative DD at this time were 830–960 (Tb = 13°C) and 900–1030 (Tb = 12.26°C).

## Discussion

*C. gnidiella* presents three generations in southern Uruguay, the same as Ringerberg *et al*. (2005) estimated for southern Brazil, while Bagnoli and Lucchi (2001) and Coscolla-Ramon (2004) mentioned three to four generations in the wine regions of Tuscany, Italy, and Cadiz, Spain, respectively. The first generation does not cause economic damage because the berries are green. However, the second generation is responsible for the majority of the damage because it coincides with berry ripening.

Pinot noir escapes economic damage even in years when the number of male moths caught was very high because it is harvested early. Similar results were observed on this cultivar by Bisotto-de-Oliveira *et al*. (2007) in Bento Gonçalves, Brazil. On Tannat, the damage is slight, depending on the year, but Gewürztraminer clusters are seriously affected at harvest time. The cultivar Gewürztraminer is over-ripened to obtain a higher-quality wine. Even in the years when Tannat and Gewürztraminer had similar male moths caught and harvest dates, damage was much higher in the latter, suggesting the insect prefers this cultivar. Plant volatiles and/or grape fermentation may act as chemical signals to the pests indicating places suitable for copulation and oviposition ( Bisotto-de-Oliveira *et al.*^2007^). In Progreso, Cabernet Sauvignon damage and catches were very low throughout the season, although this cultivar is harvested as late as Gewürztraminer. We concluded that, there was no direct relationship between male cumulative capture from December to harvest time and damage, nor was there a relationship with the maturity date of the late cultivars. Moth detection in pheromone traps enables early prediction of the start of larval feeding on clusters, but the intensity of damage is more closely related to cultivar than with adult catches.

Our results show that *C. gnidiella* does not have a winter diapause in southern Uruguay and does not require an alternate winter host; it can complete its entire life cycle in the vineyard. Ben-Shaul *et al*. (1991–1992) found similar results when they studied the overwintering of this species in avocado in Israel; larvae remained all winter in dried fruits. The low abundance of flights from the overwintering generation was probably related to the larvae mortality caused by low temperature during the winter months. Depending on the year, average temperatures were below 12°C on 52–60% of winter days. In the 2005–2006 season, spring and early summer had very low populations, despite high male moths caught at the end of the previous autumn. This resulted from a tornado that struck the area in August 2005 and killed larvae and pupae, which was confirmed by observing the remains of clusters inside the cloth bags.

According to our results, the natural parasitism does not appear as an effective measure to reduce populations. Bagnoli and Lucchi (2001), in a review of the current status of biological control in this species, reported small numbers of parasitoids in different areas of the world where the pest is widespread, although Bisotto-de-Oliveira *et al.* (2007) identified five species of parasitoid associated with *C. gnidiella* in Brazil.

Based on phenological models and population monitoring, although we use two temperatures as lower-threshold and two thermal constants, we identify three generations in both cases. Avidov and Gothilf (1960) determined the development thresholds and thermal constants from larvae fed on grapes, while Ringenberg *et al.* (2005) estimated those parameters from larvae fed an artificial diet, which could explain the different values. Nevertheless, both sets of values were suitable to estimate the onset and duration of *C. gnidiella* generations in Uruguay. The completion of the first generation was estimated with a maximum error of 2 or 3 days and the second with an error of 6 or 7 days, depending on year, regardless of the development thresholds used. Overlapping stages of development at the end of the season would explain the greater error in the second generation. The DD model could provide adequate forecasts and facilitate monitoring of insect activity in the field. Therefore, one can calculate how the pest population will develop until the harvest and take the necessary measures to prevent damage to the grapevines.

The predictive capacity of models is especially interesting when simulating population dynamics (Holt and Cheke 1997) and our models should contribute to determining the best times to implement different pest control strategies targeted at the first generation of *C. gnidiella* to prevent damage before harvest. The efficacy of pesticides is related to the percentage of emergent adults and to egg hatch, particularly in species with hidden larval stages (Butcher and Haynes 1960). Conventional control of this pest in its larval stage has not been very efficacious (Bisotto-de-Oliveira *et al.*^2007^). This model could be very useful to improve the efficacy of insect-growth-regulator pesticides, which require precisely timed applications (Ascher *et al.*^1983^), and facilitate the application of insecticides at the egg stage. However, additional data will be needed to validate the model. Analysis by cultivar, despite having no repetitions in space, allowed to verify that population growth was not affected by the specific characteristics of one cultivar. Therefore, we could analyse the pool of information to get a single model where "the cultivar" became a repetition in space. This is the first contribution of a widest line of research planned to study the spatial distribution of *C. gnidiella* in the whole area of viticulture production in Uruguay.
